# Microbial infection among SARS-COV-2-infected patients in a COVID-19-dedicated tertiary care hospital of Bangladesh: a cross-sectional study

**DOI:** 10.1099/acmi.0.000727.v3

**Published:** 2024-08-20

**Authors:** A. N. M. Shamsul Islam, Nasreen Farhana, Rafaat Choudhury, Naznin Akter Jahan, Mohammad Jamal Uddin, Md. Nazmul Hassan Refat, Fatima Nasreen, Fahmida Khanam

**Affiliations:** 1 Department of Public Health and Hospital Administration, National Institute of Preventive and Social Medicine (NIPSOM), Dhaka, Bangladesh; 2 Department of Microbiology and Mycology, National Institute of Preventive and Social Medicine (NIPSOM), Dhaka, Bangladesh; 3 Department of Nutrition and Biochemistry, National Institute of Preventive and Social Medicine (NIPSOM), Dhaka, Bangladesh; 4 Department of Parasitology, National Institute of Preventive and Social Medicine (NIPSOM), Dhaka, Bangladesh

**Keywords:** anti-HCV, dengue NS1, co-infection, COVID-19, ELISA, HBsAg, microbes, RT-PCR, SARS-CoV-2

## Abstract

**Objectives.** This study aimed to determine patterns of respiratory, blood-borne and uropathogenic microbial pathogens among SARS-CoV-2-infected patients in a COVID-19-(coronavirus disease 2019) dedicated tertiary care hospital in Dhaka, Bangladesh.

**Design.**This was a cross-sectional study.

**Setting.** In a COVID-19-dedicated tertiary care hospital in Dhaka, Bangladesh, conducted from March to June 2021.

**Participants.** Hospitalized individuals with COVID-19 infection regardless of age or sex.

**Primary and secondary outcome measures.** The percentage of co-infected COVID-19 patients and the characterization of the micro-organisms responsible for co-infection served as the primary outcome measures. Finding any associations between co-infection and age, co-infection and sex and co-infection and comorbidity was the secondary outcome variable.

**Interventions.** Not applicable.

**Results.**Out of 79 patients, 61 % were male, and the mean age was 49.53 years. Co-infection was seen in 7.7 % of patients, out of which 5.1 % of isolates were from urine samples, followed by 2.6 % from blood. Bacteria isolated from urine were *Enterococcus* (2.6 %), coagulase-negative *Staphylococcus* (CONS) (1.3 %) and *Enterobacter* spp. (1.3 %). *Pseudomonas* spp. was the only organism isolated from blood sample. Mixed growth was found in nasopharyngeal and throat swabs, with the predominant species being Staphylococcus aureus and Streptococcus spp. At the time of data collection, 55.7 % of patients had been given antimicrobials, and 30.4 % of patients had been given a single antimicrobial. HBsAg was positive in 1.3 % of patients and none were anti-hepatitis C or dengue NS1Ag positive.

**Conclusion.** Microbial infection has been seen to be associated with SARS-CoV-2 infections and is of great value in prescribing antimicrobials and reducing fatal outcomes of hospitalized patients.

## Data Summary

The supporting Excel file is uploaded as supplementary material to analyse the results of this work.

## Introduction

Coronavirus disease 2019 (COVID-19) emerged in Wuhan, China, in December 2019, caused by severe acute respiratory syndrome coronavirus-2 (SARS-CoV-2), a single-stranded RNA virus [1]. This infection threatens public health and the global economy. In light of this, the Whole Health Organization (WHO) decided to declare it a public health emergency of global significance [2]. Respiratory viruses often induce opportunistic bacterial infections. Co-infection in COVID-19 patients may be attributed to virus-induced airway injury, cellular loss, goblet cell hyperplasia, mucus secretion alterations, altered ciliary function, decreased gaseous exchange and immunological damage [3]. Acute epithelium damage triggered by respiratory viruses can allow for the infiltration of other microbes. So, concerns have been raised about co-infections with known viruses, bacteria and fungi in COVID-19 patients [4].

Co-infection with SARS-CoV-2 was observed at 20.7 %, including rhinovirus (6.9 %) and orthopneumovirus (5.2F %), by utilizing real-time reverse transcriptase-PCR (RT-PCR) [5]. Secondary infections by *Escherichia coli*, *Klebsiella pneumoniae* or *Stenotrophomonas maltophilia* caused serious consequences such as bacteremia, sepsis and nosocomial pneumonia (NP) in SARS patients [6]. Additionally, in influenza pandemics, NP was frequently linked to *Streptococcus pneumoniae*, *Haemophilus influenzae* and *Staphylococcus aureus* [7]. A prolonged stay in the intensive care unit (ICU) or higher mortality rates have been associated with severe cases of respiratory opportunistic pathogens like *Burkholderia cepacia* complex (BCC) bacteria, *Staphylococcus epidermidis* and *Mycoplasma* spp. [8]. However, it is yet unclear exactly what roles co-infections and/or superinfections in people with COVID-19 infection play [9]. The widespread use of empiric broad-spectrum antimicrobials in conjunction with SARS-CoV-2 has contributed to the emergence of multidrug-resistant micro-organisms [10].

Clinical laboratories in our country confront significant challenges in swiftly and accurately identifying infected people with potentially huge numbers of asymptomatic cases. Moreover, there is a paucity of data regarding co-infection in COVID-19 patients in Bangladesh. Evaluation of the respiratory tract microbiota linked to SARS-CoV-2 and co-infection patterns can offer helpful advice and recommendations for managing the COVID-19 pandemic’s multidrug-resistant bacterial illnesses. Keeping in view the above-stated facts, this study aimed to characterize microbial infections in COVID-19-infected patients as evaluating SARS-CoV-2-associated microbial infections will help to combat the COVID-19 pandemic. Uncertainty still exists regarding the microbial makeup of the respiratory tract, urinary tract and other infected tissues, as well as their potential pathogenic contributions to the various levels of disease severity in COVID-19 patients. To enhance preventive care and lower mortality and morbidity among hospitalized patients, infection detection and tracking are absolutely essential.

By analysing patterns of respiratory, blood-borne and uropathogenic bacteria in a COVID-19-dedicated hospital in Bangladesh, this study sought to define microbial co-infection among SARS-CoV-2-infected patients.

## Methodology

### Study design

A cross-sectional study.

### Setting

This study was conducted at Microbiology Laboratory of National Institute of Preventive and Social Medicine (NIPSOM), Mohakhali, Dhaka, Bangladesh, which is the apex institution in the field of public health and has important role and potential in conducting research on national and international health issues usually through sponsorship of Bangladesh Medical Research Council, the WHO and other national and international agencies/funding authorities.

### Study place

Kurmitola General Hospital (KGH).

Capacity and facilities: KGH is a 500-bedded hospital located in Cantonment area, Dhaka, which has outdoor and indoor services including Departments of Surgery, Medicine, Gynaeclology, Ophthalmology, Ear Neck, and Throat (ENT), Paediatric, Orthopedics, Dentistry, and Pulmonology. Considering the infrastructure, location and other facilities, the Ministry of Health and Family Welfare of Bangladesh has dedicated this hospital to admit and treat epidemic/pandemic cases like SARS-CoV-2.

### Study period

NIPSOM conducted this study from March to June 2021 on 79 COVID-19 patients diagnosed using a convenient sampling method and admitted in KGH, a COVID-19 dedicated tertiary care hospital in Dhaka, Bangladesh, and May to June 2021 served as the data collection period. Clinical and sociodemographic information on the patient was taken from their history and follow-up sheet.

### Participants

Irrespective of age and sex, all hospitalized SARS-CoV-2-infected patients diagnosed according to the National Guidelines on Clinical Management of COVID-19 were selected conveniently for this study [11]. Due to altered mental consciousness, it would be difficult to get informed written consent and collect relevant samples from critically ill and mentally unstable patients, so they were excluded from the study.

### Variables

- Proportion of co-infection in COVID-19 patients: the percentage of the co-infected COVID-19 patients
- Patient characteristics: age, sex, religion and comorbidity of diagnosed COVID-19 patients enrolled in this study
- Type of infection: the presence or absence of blood stream infection (bacterial or viral) or UTI in diagnosed COVID-19 patients enrolled in this study
- Use of antimicrobials: frequency of single or multiple antimicrobial administered in diagnosed COVID-19 patients enrolled in this study collected from hospital records

### Diagnostic criteria

- Co-infection: it is the term used to describe when two or more pathogen species and/or strains simultaneously infect a cell or host.
- COVID-19 patient: in accordance with the National Guidelines on Clinical Management of COVID-19, a confirmed case is a person with laboratory confirmation of COVID-19 infection either by RT-PCR or Immunochromatographic test (ICT) or both, irrespective of clinical signs and symptoms [11].
- Urinary Tract Infection (UTI): patients were identified as having UTIs if their centrifuged urine sample met the criteria of 5 pus cells per high-power field (HPF) in urine microscopy and a colony count of >10 5 c.f.u. ml^−1^ in urine culture.
- Blood stream infection (BSI): patients with positive blood culture and/or serological assay results were diagnosed as having BSI.

### Data sources and measurement

The patient’s socio-demographic and clinical data were extracted from their history and follow-up sheet. Biological samples such as respiratory specimens (nasopharyngeal and throat swabs), blood and urine were collected, maintaining strict aseptic precautions for COVID-19 patients. Each sample tube was appropriately labelled with the patient’s name, ID, date and time of collection and transported to the NIPSOM Microbiology laboratory in Mohakhali, Dhaka, Bangladesh.

### Microbiological examination

Nasopharyngeal swab (NS) and throat swab (TS) were cultured onto blood agar and MacConkey’s agar media, uncentrifuged urine sample onto HiCrome UTI agar media using a sterile calibrated wire loop (0.002 ml) and blood onto brain heart infusion broth cultures separately as soon as possible and incubated at 37 °C in aerobic condition for 24 h, according to the standard microbiological procedure. The isolated organisms were identified by their colony form, pigment production, hemolytic criteria in culture, staining features under microscopy and pertinent biochemical tests after 24 h of inoculation. The centrifuged urine deposit was examined microscopically for significant pus cells (>5/HPF), RBC, epithelial cell, cast and crystal and biochemical tests (glucose, albumin) were undertaken. Serum was separated from the blood in corresponding labelled Eppendorf tubes by centrifugation at 2500 r.p.m. for 15 min, then tested for serological tests [ICT for Dengue NS1 antigen and HBsAg test for hepatitis B virus (HBV) and anti-HCV test for hepatitis C virus (HCV) by ELISA] and stored at −20 °C refrigerator.

### Quality control measures

Quality control measures taken to ensure the accuracy and reliability of the results include a training before the start of the data collection to instruct all personnel related to the study regarding the participant enrollment, collection and entry of data, maintaining standard operating procedure during all stages of data collection, sample collection and processing, using of control ATCC strain for bacterial identification maintaining confidentiality in all stages.

### Efforts to address potential sources of bias

By developing a comprehensive research plan, we keep in mind the possibility of bias in every step of the research study planning process. The participants are selected for a sample based on convenience or who volunteers rather than equal probability. This can lead to a sample that is not representative of the larger population, and the findings may not apply to other groups. A member of the institutional review board examined the research protocol. All research data were recorded anonymously and in detail. Pretesting and an extensive literature review were used in this study.

### Study size

Seventy-nine COVID-19-infected hospitalized patients conducted were selected by non-probability (convenience) sampling from March to June 2021. During our study period, the probability sample survey would have been too slow or too infrequent to meet the required data demand. So, the non-probability sampling method was applied.

### Statistical analysis

The Statistical Package for Social Sciences computer program version 23 was used to evaluate data that had been placed into an Microsoft Excel spreadsheet. For handling missing data, there had been applied the most common approach that simply omitted the case with the missing data and analysed the remaining data. Descriptive statistics were applied to all variables, and the results were presented as frequencies and percentages. Mean and sd were used in descriptive analysis for numerical variables. The use of descriptive and analytical statistics, such as the Fisher’s exact test and the chi-square test, was made. When *P*<0.05, all analyses were deemed significant.

### Patient and public involvement

We created a study question about SARS-CoV-2 and co-infection detection in accordance with Bangladesh’s needs in terms of public health. No patients or members of the general public were engaged in the study’s recruiting, execution or reporting. The study findings will be disseminated to all through publication in a peer-reviewed open-access journal, so that this research may contribute further by being a part of a meta-analysis in the future.

## Results

Sociodemographic data and important clinical examination findings of all 79 patients were obtained from hospital records, but microbiological analysis was done from samples collected from 78 patients. Out of 79 patients, the age ranged from 17 to 86 years (mean 49.53±SD 14.82 years); the highest, 53 %, were below 50 years of age, and 61 % were male. The most frequent comorbidities were diabetes mellitus (31.6 %), hypertension (29.1 %) and bronchial asthma (13.9 %). Out of the 79 hospitalized patients, 55.70 % of patients received antimicrobials and 25.3 % of them received agents with broad‐spectrum coverage by amoxicillin–clavulanic acid, azithromycin and doxycycline combination with or without ivermectin. During data collection time, 30.4 % of patients received single antimicrobials, such as ceftriaxone, meropenem, ivermectin, azithromycin and cefixime (Table 1).

**Table 1.** Background and clinical characteristics of respondents

Basic features		Frequency (%)
**Age** (range: 17–86 years)	Below 50 years	42 (53.2)
(Mean: 49.53 years±SD 14.82 years)	Above 50 years	37 (46.8)
**Sex**	Male	48 (60.8)
**Religion**	Muslim	77 (97.5)
**Having symptoms**		
Fever>38 °C		40 (51.9)
History of fever within 14 days		43 (54.5)
Cough		40 (50.6)
Running nose		17 (21.5)
Respiratory distress		30 (40.0)
Diarrhoea		8 (10.1)
Vomiting		4 (5.1)
**Having comorbities**		
Chronic obstructive pulmonary diseases		7 (8.9)
Diabetes mellitus		25 (31.6)
Hypertension		23 (29.1)
Chronic heart diseases		4 (5.1)
Bronchial asthma		11 (13.9)
Chronic kidney diseases		3 (3.8)
Cardiovascular diseases		6 (7.6)
History of acute myocardial infection		2 (2.5)
History of stroke		2 (2.5)
**Use of antimicrobials**		
Single antimicrobial		24 (30.4)
Multiple antimicrobial		20 (25.3)
No use of antimicrobials		35 (44.3)

Of them, biological specimen was collected from 78 COVID-19-infected patients where females had an infection rate of 2.56 % for blood stream infections and 1.28 % for UTIs, while males only had an infection rate of 3.84 % for UTIs. Among 78 patients, the infection rate was 5.13 % with respect to those below 50 years (Table 2).

**Table 2.** Microbial infection on the basis of age and sex among COVID-19-infected patients (*n*=78)

Characteristics		Blood Stream infection (bacterial), f (%)	Blood Stream infection (viral), f (%)	Urinary tract infection (UTI), f (%)
Sex	Male	0	0	3 (3.84)
	Female	1 (1.28)	1 (1.28)	1 (1.28)
Age	Below 50 years	1 (1.28)	0	3 (3.84)
	Above 50 years	0	1 (1.28)	1 (1.28)

Out of 78 patients, co-infection was seen in 7.7 % of patients, out of which 5.1 % of isolates were from urine samples, followed by 2.6 % from blood (Table 3).

**Table 3.** Microbial infection among COVID-19-infected patients (*n*=78)

Type of infection	Microbial infection		
	Present, f (%)	Isolated organism	Absent, f (%)
Blood stream infection (bacterial)	1 (1.3)	*Pseudomonas* spp.	77 (98.8)
Blood stream infection (viral)	1 (1.3)	Hepatitis B virus	77 (98.8)
Urinary tract infection (UTI)	4 (5.1)	*Enterobacter* spp. (1)Coagulase-negative *Staphylococcus* spp. (1)*Enterococcus* spp. (2)	74 (95.0)
**Total co-infection found in 6 (7.7 %) patients**			

Urine analysis could not be done from one patient due to insufficient quantity and so the case with the missing data was omitted during the analysis of urine sample, but all other data analysis was done on enrolled 79 patients. Routine microscopic examination of the urine of 77 COVID-19 patients revealed the presence of pus cells (≥ 5/HPF) in 18 % of cases, along with RBC in 6.49 % of cases. Mixed growth was found in 14.2% of urine cultures and *Enterococcus* spp. (2.6%), coagulase-negative *Staphylococcus* spp. (1.3%) and *Enterobacter* spp. (1.3%) were isolated as single organism accounted for the urinary tract infection (Table 4).

**Table 4.** Routine examination findings of urine of COVID-19 patients (*n*=77)

A. Biochemical findings:		
a. Urinary glucose status of the patients		
**Status**	**Frequency**	**Percent**
Nil	60	77.9
250 mg dl^−1^	3	3.9
500 mg dl^−1^	7	9.1
1000 mg dl^−1^	5	6.5
2000 mg dl^−1^	2	2.6
**b. Urinary albumin protein status of the patients**		
**Status ofurinaryalbuminprotein**	**Frequency**	**Percent**
Nil	69	89.6
Trace	7	9.1
One plus (≥30 mg dl^−1^)	1	1.3
**B. Routine microscopic examination findings**		
**Urine microscopy finding**	**Frequency**	**Percent**
Pus cell (≥ 5/HPF)	14	18
Pus cell (≤ 5/HPF)	63	82
RBC	5	6.49
**C. Findings of urine culture**		
**Name of the organism**	**Frequency**	**Percent**
*Enterococcus*	2	2.60
*Enterobacter*	1	1.30
Coagulase-negative *Staphylococcus*	1	1.30
Mixed growth	11	14.28
No growth	62	80.52
**Total**	77	**100**

*Pseudomonas* spp. (1.3 %) and *Hepatitis B virus* (1.3 %) were detected from the bloodstream infection. Mixed growth in blood agar and MacConkey’s agar media from 3.85 % NS and 12.82 % TS was found, whereas 14.2 % of urine cultures had no definite predominance. Among the 78 serum samples, none were positive for dengue NS1 antigen and antibody against HCV, but 1.3 % of them was found to be HBsAg positive (Table 5).

**Table 5.** Findings of nasal swab, throat swab, blood culture and serology (*n*=78)

**Culture findings**	Nasopharyngeal swab culture	Throat swab culture		
	**f (%**)	f (%)		
*No growth*	60 (76.92)	30 (38.46)		
Mixed growth	3 (3.85)	10 (12.82)		
*S.aureus*	7 (8.97)	2 (2.56)		
Coagulase-negative *Staphylococcus*	5 (6.41)	2 (2.56)		
*Streptococcus*	3 (3.85)	23 (29.50)		
*E. coli*	**0**	6 (7.69)		
*Klebsiella*	**0**	2 (2.56)		
*Pseudomonas*	**0**	3 (3.85)		
**Bacterial profile of mixed growth isolated from throat swab (*n*=10**)				
**Name of the organism**	**Frequency**	**Per cent**		
*S.aureus* and *Streptococcus* species	4	40.0		
*Streptococcus*, *E. coli*, *Pseudomonas*	2	20.0		
Coagulase-negative *Staphylococcus *and *Klebsiella*	1	10.0		
*Klebsiella*and *Acinetobacter*	1	10.0		
*Streptococcus* species, *Candida *and *E. coli*	1	10.0		
*S.aureus,Streptococcus species*and *Pseudomonas*	1	10.0		
**Total**	10	100		
**Blood culture findings**	**Frequency**	**Per cent**		
No growth	77	98.7		
*Pseudomonas* spp.	1	1.3		
**Serological test (** ***n*** **=78)**				
**Status**	**Serum HBsAgf (%)**	**Anti-HCV antibodyf (%)**		**Dengue NS1f (%)**
Positive	1 (1.3)	0		0
Negative	77 (98.7)	78 (100)		78 (100)

There was no significant association between co-infections and sociodemographic characteristics of the participants (Table 6).

**Table 6.** Association of co-infection with patients’ characteristics

**Sociodemographic characteristics**		**Association of co-infection**		***P*-value**
		**Absent**	**Present**	
Sex	Female (*n*, 31)	29 (93.5 %)	2 (6.5 %)	0.561
	Male (*n*, 48)	44 (91.7 %)	4 (8.3 %)	
Religion	Muslim (*n*, 77)	71 (92.2 %)	6 (7.8 %)	0.853
	Hindu (*n*, 2)	2 (100.0 %)	0 (0.0 %)	
Diabetes mellitus (DM)	No DM (*n*, 54)DM (*n*, 25)	51 (94.4 %)22 (88.0 %)	3 (5.6 %)3 (12.0 %)	0.281
Bronchial asthma	No (*n*, 68)Yes (*n*, 11)	63 (92.6 %)10 (90.9 %)	5 (7.4 %)1 (9.1 %)	0.606
Hypertension	No (*n*, 56)Yes (*n*, 23)	53 (94.6 %)20 (87.0 %)	3 (5.4 %)3 (13.0 %)	0.233
Total (*n*, 79)		73 (92.4 %)	6 (7.6 %)	

## Discussion

In this study, 79 COVID-19 confirmed cases in accordance with the National Guidelines on Clinical Management of COVID-19, hospitalized at KGH, Cantonment in Dhaka from March to June 2021, were included. The participants were selected based on convenience or who volunteers rather than equal probability which might lead to a sample that is not representative of the larger population, and the findings may not apply to other groups. Due to altered mental consciousness, it would be difficult to get informed written consent and collect relevant samples from critically ill and mentally unstable patients, so they were excluded from the study.

### Co-infection

In our study, bacterial and viral co-infections were found in 6 (7.7 %) COVID-19 patients; the causative organisms were *Pseudomonas* spp., hepatitis B virus, *Enterobacter* spp., coagulase-negative *Staphylococcus* spp. and *Enterococcus* spp. A meta-analysis of 6639 publications comprised a total of 118 investigations was done on the prevalence and outcomes of co-infection and superinfection with SARS-CoV-2 and other pathogens. Pooled prevalence of pathogen type stratified by co-infection was viral co-infections 10 % (6–14 %), bacterial co-infections 8 % (5–11 %) and fungal co-infections 4 % (2–7 %) which were not in line with our study findings [12].

A meta-analysis was done in Canada to determine the prevalence of bacterial co-infection (at presentation) and secondary infection (after presentation) in patients with COVID-19. Bacterial co-infection (estimated on presentation) was identified in 3.5 % of patients. The overall proportion of COVID-19 patients with bacterial infection was 6.9 %. The researchers of the meta-analysis concluded that bacterial co-infection is relatively infrequent in hospitalized patients with COVID-19 which is in accordance with our study findings [13].

In a study conducted on 99 COVID-19-positive individuals in China, 1 and 4 % had bacterial and fungal co-infections, respectively. In one patient, culture showed mixed growth of *Acinetobacter baumannii*, *K. pneumoniae* and *Aspergillus flavus*. *Candida glabrata* and *Candida albicans* accounted for 25% and 75 % of fungal infections, respectively [13], which was not similar to our study findings. However, according to a literature survey conducted in Bangladesh, *S. pneumoniae*, *S. aureus*, *K. pneumoniae*, * H. influenzae*, *Mycoplasma pneumoniae*, *A. baumannii*, *Legionella pneumophilia* and *Chlamydia pneumoniae* are the most common SARS-CoV-2 co-pathogens, followed by influenza, coronavirus, rhinovirus, enterovirus, Parainfluenza, metapneumovirus, influenza B virus, and human immunodeficiency virus [4]. However, in our study, we were unable to do these tests due to not having the resources necessary to perform cultures for fastidious anaerobic, atypical bacteria and fungi. In a study conducted on 99 COVID-19-positive individuals in China, 1 % and 4 % had bacterial and fungal co-infections, respectively. In one patient, culture showed mixed growth of *A. baumannii*, *K. pneumoniae* and *A. flavus*. *C. glabrata* and * C. albicans* accounted for 25% and 75 % of fungal infections, respectively [14].

### Urine microscopy and culture findings

In this study, regardless of urine microscopy findings, all urine samples were cultured to determine the presence of asymptomatic bacteriuria and co-infection. The findings of urine microscopy revealed that 18 % had findings suggestive of UTI (pus cell 5/HPF with or without RBC), and the remaining 82 % had cell numbers within the normal range. The majority of urine samples (79.7 %) yielded no growth in culture. All hospitalized patients were on broad-spectrum antibiotics before hospitalization or caused by fastidious organisms, which require particular growth media and conditions, which may explain this. Sterile pyuria can occur in patients with fever, certain diseases or after vigorous exercise [15]. Urine samples from just 5 individuals contained RBC, while 14 patients had pus cells (5/HPF). Interestingly, 62 (80.5 %) had no growth in culture, whereas 11 (14.2 %) showed negligible (colony count <105 c.f.u. ml^−1^) polybacterial proliferation. Four patients were found to have UTIs based on the presence of monobacterial growth (colony count ≥105 c.f.u. ml^−1^) of *Enterococcus* spp., *Enterobacter* spp. and coagulase-negative *Staphylococcus* spp. in urine cultures. The most likely reason for a negative urine culture is a urinary tract infection caused by fastidious organisms, such as *Neisseria* species, which require particular growth media and conditions. *E. coli*, *Enterococcus* spp., *Enterobacter* spp., coagulase-negative *Staphylococcus* spp. and *S. aureus* were the bacteria recovered from mixed culture. It could not be determined if the presence of these pathogenic bacteria was a result of contamination or an indication of early colonization. The effects of SARS-CoV-2 infection on lower urinary tract function were investigated in a prospective multi-centre, observational study that was carried out in two hospitals on 238 COVID-positive patients. Out of 238 patients, 122 had a bacterial urine culture, and 50 (41 %) of those patients had a positive result. *E. coli*, *K. pneumoniae* and *Proteus* spp. were prevalent uropathogens. About 54 % of COVID-19 patients had WBC in their urine [16]. Their study findings are similar to our study, where most urine cultures were negative.

### Nasopharyngeal and throat microbiota

This study revealed that the nasopharyngeal and oropharyngeal microbiomes of COVID-19 patients exhibited mixed growth on blood agar and MacConkey's agar media, with a prevalence of 3.9% of NS and 12.8% of TS. Gram-positive bacteria (*Staphylococcus aureus* and *Streptococcus* spp.) were the predominant (40 %) isolated from the mixed growth of throat swab cultures, and in order to ascertain if the presence of the virus in the nasopharynx was related to alterations in the local microbiota, De Maio et al. at the IRCCS Hospital in Rome, Italy, investigated the bacterial communities of SARS-CoV-2 in infected and uninfected patients. Their research revealed that there were no changes in the composition of the microbiota in response to SARS-CoV-2, which is consistent with our discovery that the nasopharynx microbiota of SARS-CoV-2 patients was identical to that of healthy people [17]. However, in a study in China done for the characterization of respiratory microbiota in COVID-19 patients, the predominant respiratory microbial taxa of severely ill patients were BCC, *S. epidermidis* or *Mycoplasma* spp. [8].

### Blood-borne pathogen

The opportunistic pathogen that forms biofilms in COVID-19 patients, *Pseudomonas aeruginosa*, aggravates their medical conditions. Only *Pseudomonas* spp. was found to be the cause of bloodstream infections in our study’s COVID-19 participants. *P. aeruginosa* is the second most often found infection in COVID-19 patients, according to [18]. According to a study conducted in China, *P. aeruginosa* undergoes complicated genetic modifications when SARS-CoV-2 is present in order to boost its antimicrobial resistance, maintain its colonization and cause sickness [19]. Contrary to our findings, among 750 COVID-19 ICU patients in India, *A. baumannii* was the most common isolate (32.8 %), followed by *K. pneumoniae* (21.9 %) [20]. There are two possible causes for the low rate of organism detection in blood cultures revealed in our investigation. First off, our BSI sample was smaller than in other studies, which led to a less trustworthy assessment of the prevalence of the causal agent. Second, the majority of COVID-19 patients received broad-spectrum antibiotic therapy since it was challenging to distinguish between primary viral pneumonia and bacterial pulmonary superinfection.

### Viral co-infection

Due to similar symptoms and inadequate diagnostic resources, co-infection with dengue and COVID-19 is challenging to diagnose in tropical areas like Bangladesh. Only two incidences of COVID-19 and dengue co-infection were found in an investigation of 123 COVID-19 patients and 183 dengue patients in Cali, Colombia [21]. Five of the 20 COVID-19 patients admitted to Holy Family Hospital’s critical care unit (ICU) in Rawalpindi, Pakistan, also tested positive for dengue virus serotype-2 [22]. However, none of the COVID-19 patients in our study had a positive dengue virus test. The reduced onset of clinical dengue in subclinically or slightly ill SARS-CoV-2 patients may have been caused by viral interference that prevented dengue entry and replication. [23]. According to various studies conducted in China, the prevalence of HBV in COVID-19 patients ranged from 0.8 to 12.2 %, which is not consistent with the results of our investigation, which found a 1.3 % co-infection rate [24]. From 1 December 2019 to 9 August 2020, researchers in Iran conducted a systematic study to compile the data on COVID-19 patients with HBV or HCV co-infections. They searched several electronic databases and preprint servers. Of the 941 data found, 235 patients with COVID-19 had HBV and 22 had HCV infections [25]. All of the COVID-19 patients were examined for HCV infection as part of our trial, and none tested positive for anti-HCV. The results of our study confirm the generally decreased prevalence of HBV and HCV in Bangladesh’s general population. In our nation, the overall prevalence of HBV is estimated to be 5.5 % [26]. The amount of the HCV in Bangladesh is not well known. According to published literature, the prevalence rates in the general population range from 0.2 to about 1 %. In Bangladesh, the extended programme of immunization schedule for infants first included a mass vaccine against HBV in 2003, with a coverage rate of more than 97 %. As a result, there has been progress because the prevalence has decreased [27].

## Conclusion

Gram-negative organisms were the most typical type of organism detected, even though the overall number of COVID-19 patients with co-infection was low among hospitalized patients in our study group. It is important to understand how co-infection affects the severity of COVID-19 and treatment outcomes for effective antimicrobial therapies. However, the study is limited by its sample size which may not be representative of the larger population of COVID-19 patients. The study relies on hospital records for data collection, which may not provide a complete and accurate picture of the patients’ clinical status and co-infections. Certain tests for specific pathogens and identification of eco-genomic characteristics or genes linked to antimicrobial resistance of bacterial isolates could not be conducted due to a lack of resources. Prospective research is required to determine how COVID-19 patients’ microbiological co-infections affect efforts to combat antibiotic resistance.

## Recommendation

- Additional large-scale prospective studies are required to confirm the prevalence of bacterial and fungal infections and how they affect COVID-19 outcomes.
- Next-generation sequencing and PCR are highly sensitive methods for detecting possible co-infecting micro-organisms with SARS-CoV-2.
- Improvements in the detection of bacterial and fungal infections among COVID-19 patients, a decrease in the overuse of antibiotics in low-risk locations and support for timely access to medication are all important components of antimicrobial stewardship programmes.

## supplementary material

10.1099/acmi.0.000727.v3 Uncited Supplementary Material 1.

## Abbreviations

BCC: *Burkholderia cepacia* complex
BSI: blood stream infection
COVID-19: coronovirus disease 2019
HBV: hepatitis B virus
HCV: hepatitis C virus
HPF: high-power field
ICU: intensive care unit
KGH: Kurmitola General Hospital
NIPSOM: National Institute of Preventive and Social Medicine
NP: nosocomial pneumonia
NS: nasopharyngeal swab
RT-PCR: real-time reverse transcriptase-PCR
RT-PCR: real-time reverse transcriptase-PCR
SARS-CoV-2: severe acute respiratory syndrome coronavirus-2
TS: throat swab
WHO: World Health Organization
